# Equine infectious anemia virus in China

**DOI:** 10.18632/oncotarget.20381

**Published:** 2017-08-21

**Authors:** Hua-Nan Wang, Dan Rao, Xian-Qiu Fu, Ming-Ming Hu, Jian-Guo Dong

**Affiliations:** ^1^ Department of Veterinary Medicine, College of Animal Sciences, Zhejiang University, Hangzhou, China; ^2^ School of Animal Husbandry and Medical Engineering, Xinyang Agriculture and Forestry University, Xinyang, China; ^3^ Guangdong Key Laboratory of Laboratory Animals, Guangdong Laboratory Animals Monitoring Institute, Guangzhou, China; ^4^ Shanxi Provincial Animal Disease Control Center, Taiyuan, China; ^5^ The First Affiliated Hospital of Harbin Medical University, Harbin, China

**Keywords:** equine infectious anemia virus, epidemic, vaccine, virulence, immune response

## Abstract

Equine infectious anemia is an equine disease caused by equine infectious anemia virus, which was first reported in 1840. Equine infectious anemia virus research in China started in the 1960s, focusing on etiology, pathology, diagnosis, and immunology. Notably, in 1978 an attenuated vaccine was successfully developed for equine infectious anemia virus, effectively preventing equine infectious anemia virus in China. This article will review equine infectious anemia virus in China, including past and recent research, and commemorate scientists who have made great contributions to equine infectious anemia virus prevention.

## INTRODUCTION

Equine infectious anemia virus (EIAV) is a member of the *Lentivirus* genus of the *Retroviridae* family. The *Lentivirus* genus also includes human immunodeficiency virus (HIV), simian immunodeficiency virus (SIV), feline immunodeficiency virus (FIV), bovine immunodeficiency virus (BIV), and Maedi-visnavirus (MVV) EIAV almost infected equids worldwide. It causes a persistent infection characterized by recurring febrile episodes associated with viremia, fever, thrombocytopenia, and wasting symptoms [1]. EIAV raised great concern among the international veterinary community due to the serious economic losses it caused, as well as the unique nature of this pathogen. In China, scientists successfully controlled the spread of EIAV, especially when an effective vaccine was widely applied. Here, we will focus on the control of EIAV in China.

### EIAV introduction and epidemic in China

Reports and records of EIAV did not appear in China until the early 20th century. Japan spread EIAV to China during World War II. Due to an incomplete quarantine, horses with a latent infection were traded from the former Soviet Union and Mongolia, which also introduced EIAV to China. From 1954 to 1959, EIAV mainly occurred in Heilongjiang, Jilin, Liaoning, Inner Mongolia, Yunnan, Hebei, and the Shanxi province. EIAV prevalence in Shandong, Gansu, Jiangsu, and the Anhui Province was due to infected horses purchased from Xinjiang. EIAV prevalence in Hebei, Beijing, Tianjin, Shaanxi, and Henan provinces was caused by EIAV-latent horses purchased from Liaoning, Jilin, and Heilongjiang. The Chinese EIAV epidemic was started by horses traded from foreign countries, and gradually spread from north to south, eventually inducing a nationwide outbreak.

**Figure 1:**
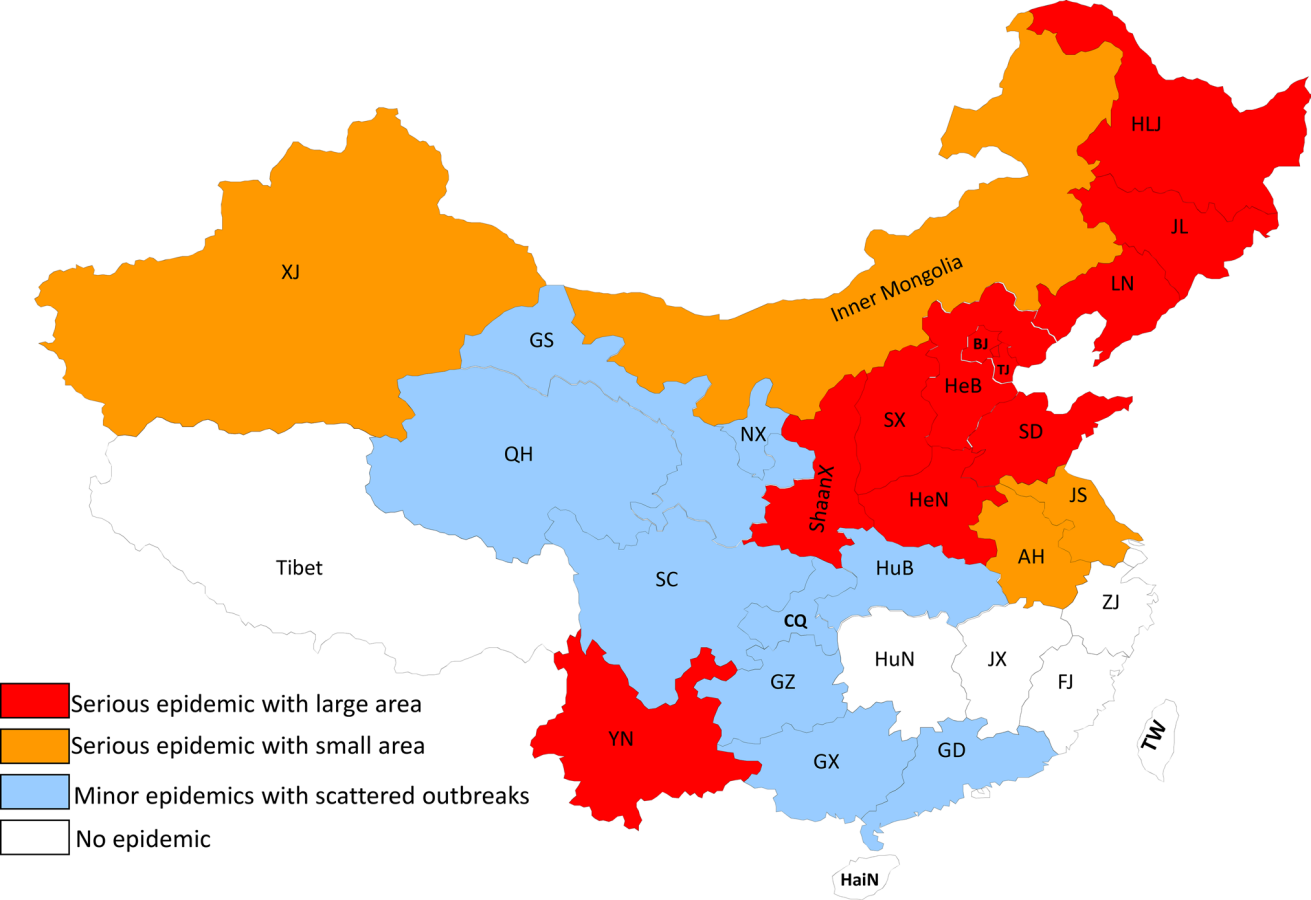
EIAV epidemic in China from 1954 to 1990 The EIAV epidemic state in China from 1954 to 1990 is summarized. The different colors represent epidemic degree of EIAV in different provinces. The jacinth represents serious epidemic with large area; the brown represents serious epidemic with small area; the light blue represents minor epidemics with scattered outbreaks.

From 1954 to 1989, EIAV broke out in 23 provinces to varying degrees, contaminating 77% of the country’s total provinces (Figure 1) [2]. In the 1950s, EIAV was reported in only nine provinces: Heilongjiang, Inner Mongolia, Yunnan, Guizhou, Jilin, Guangdong, Guangxi, Hebei, and Shanxi. During the 1960s, the epidemic spread to 10 more provinces for a total of 19, including Liaoning, Beijing, Xinjiang, Shandong, Tianjin, Shaanxi, Henan, Gansu, Jiangsu, and Anhui. EIAV was reported in two more provinces in the 1970s, Qinghai and Sichuan, raising the total to 21 provinces. EIAV expanded into Ningxia and Hubei in the 1980s, capping the total at 23 provinces. Among all these regions, Heilongjiang, Liaoning, Jilin, Inner Mongolia, Hebei, Henan, Tianjin, Beijing, Shandong, Shanxi, Shaanxi, and Yunnan were the most infected [2]. The Chinese EIAV epidemic was stimulated by four factors: importing infected animals, frequent movement and trading of infected horses without effective control, no early detection method at early stage, and not sterilizing medical devices [2].

### EIAV control in China

EIAV prevention and control is accompanied by the development of detection technology. Because quarantine was the only control measure available in the early stages of EIAV, clinical comprehensive diagnosis (CCD) was widely applied [2]. EIAV could not be confirmed according to just one of the CCD methods, which included epidemiological investigation, clinical, blood, and pathology examination. CCD was used to screen possibly infected horses from 1954 to 1973 in China. EIAV-infected horses were quarantined or slaughtered to prevent spread of the virus. However, CCD could not detect latent infection which led to the reemergence of EIAV.

From 1974 to 1978, two serological methods dependent on antibody detection were developed to battle EIAV. The first was the complement-fixation (CF) test which detected specific antibodies that fixed complement when interacting with the corresponding antigen. Hemolysis was used as an indicator system to detect residual complement [2]. Another serological test based on agar gel immunodiffusion (AGID) [2]. EIAV-seropositive animals were either euthanized or kept inquarantine for the rest of their life, depending on local regulations. The use of these two tests controlled the EIAV infection in China. Not only were CCD, CF, and AGID time consuming and difficult to interpret, but some infected horses were misdiagnosed because some animals are positive for only one of the two serological tests. These disadvantages were overcome in 1979 when an enzyme-linked immunosorbent assay (ELISA) was developed to detect EIAV antibody [3], which was simple, quick, sensitive, and specific.

**Figure 2:**
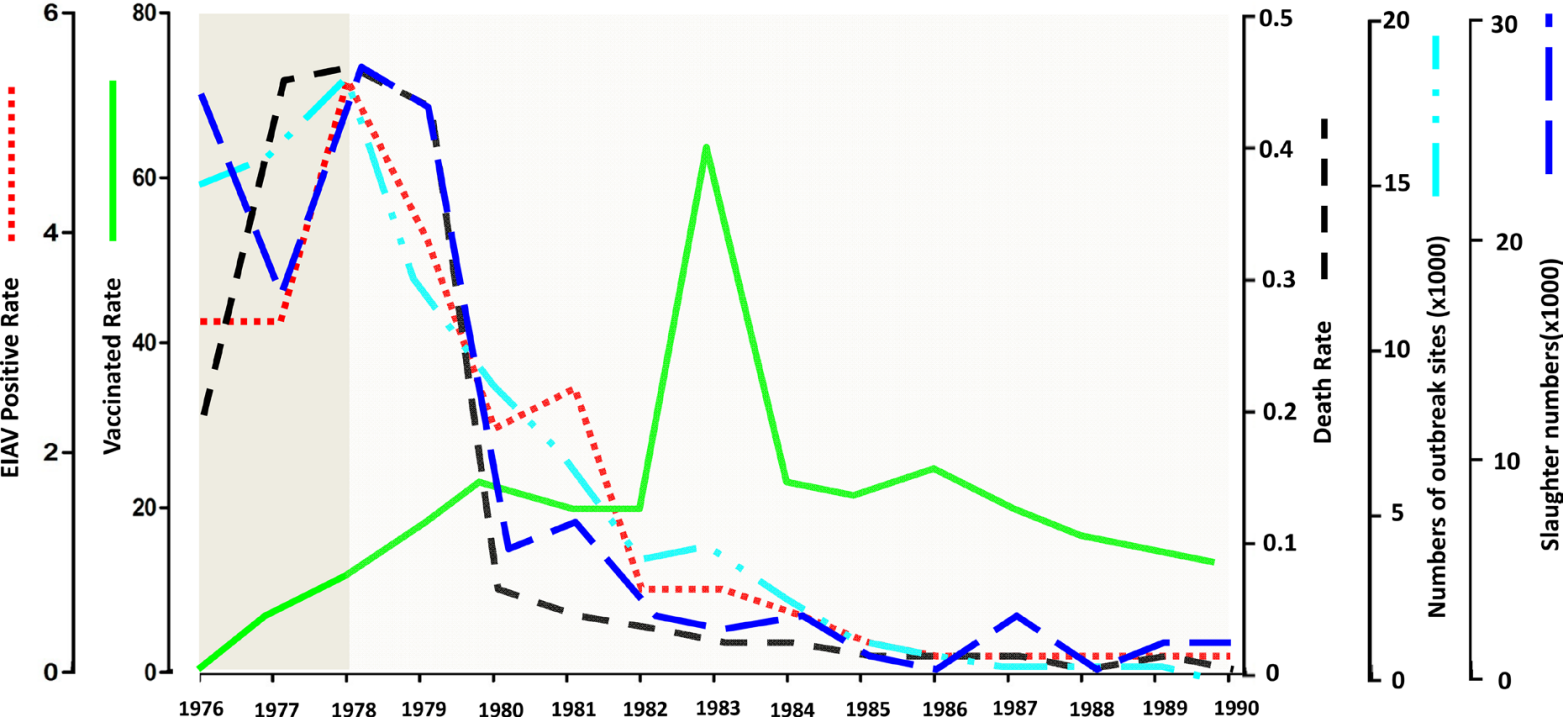
Statistics of EIAV control in China from 1976 to 1990 The EIAV control in China from 1976 to 1990 is summarized. The red dotted line represents EIAV positive rate, the green line represents vaccinated rate; the black dotted line represents death rate; the wathet dotted line represents numbers of outbreak sites; the deep blue dotted line represent slaughter numbers.

It is impossible to eradicate EIAV merely with quarantine measures. Antibody levels are not constant in the infected animal, so it is not possible to detect all the subclinical cases. An integrated control program including vaccination and quarantine was adopted. This not only eliminated most of the infected animals and reduced EIAV spread, but also enabled most of the healthy horses to obtain immunity. These measures accelerated the control and eradication of EIAV in China (Table 1; Figure 2).

**Table 1:** Statistics of EIAV control from 1976 to 1990 in China

Year	Horse numbers (Ten thousands)	Detected numbers	Positive numbers	Positive rate (%)	Death numbers	Death Rate (%)	Slaughter numbers	Vaccinated numbers	Vaccinated Rate (%)	Outbreak sites
1976	1862.53	2743166	87760	3.2	35063	0.19	26814	12476	0.067	15236
1977	1870.79	2525111	79541	3.15	81732	0.44	18010	1156245	6.181	15952
1978	1830.01	2096203	111117	5.3	81994	0.45	27790	1992940	10.89	18410
1979	1913.01	2743157	107623	3.92	80168	0.42	26396	3358207	17.555	12433
1980	1989.26	1695238	37084	2.19	12002	0.06	6496	4409572	22.167	9086
1981	2053.35	1330194	33120	2.5	8351	0.041	7348	3908099	19.033	7006
1982	2070.28	2141242	14895	0.7	6078	0.029	3562	3952403	19.091	4029
1983	2062.36	1588637	12257	0.77	4094	0.020	2398	13251864	64.256	4169
1984	2126.73	2074567	11665	0.56	3947	0.019	3086	4813241	22.632	2525
1985	2174.75	1486474	4168	0.28	2547	0.012	1449	4446776	20.447	1242
1986	2181.08	1725410	2610	0.15	1676	0.008	798	5225377	23.958	962
1987	2156.53	1754116	1728	0.1	838	0.004	3191	4173862	19.355	638
1988	2155.13	1317615	2195	0.16	405	0.0018	1049	3549881	16.472	369
1989	2118.24	1334831	1669	0.12	561	0.0026	2097	3178578	15.006	298
1990	2076.39	1345772	820	0.06	108	0.0005	1801	2663679	12.828	36
Sum		27901733	508252		319564		132285			92391

### EIAV live attenuated vaccine development

For more than a century, the most successful vaccines were live attenuated vaccines which have been widely used to control the epidemics of many viruses, such as smallpox, polio, and measles [4, 5]. Since the 1960s,EIAV brought great losses to Chinese agriculture. After nearly 20 years of effort, Chinese scientists developed an effective live attenuated EIAV vaccine protected horses not only against homologous EIAV strains, but some heterogeneous virus strains as well [6–8]. The Chinese live attenuated EIAV vaccine is the only large-scale lenti-viral vaccine in the world, and serves as a model for the development of other lenti-virus vaccines [9].

**Figure 3:**
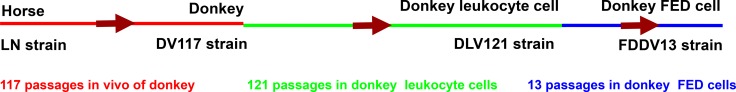
Development of Chinese EIAV live attenuated vaccine The development of Chinese EIAV live attenuated vaccine is summarized. EIAV_LN_:LN strain serial 16 passages in horse; EIAV_DV117_: LN strain serial 117 passages in donkey; EIAV_DLV121_:DV117 strain serial 121 passages in donkey leukocyte; EIAV_FDDV13_:DLV121 strain serial 13 passages in fetal donkey dermal cell.

Chinese scientists isolated a wild highly-pathogenic strain from horses in Liaoning, EIAV_LN_, and attempted to adapt this virus to several other animals, tissues, or cell lines. All these attempts failed, except in donkey. The virus experienced 117 passages (EIAV_DV117_) in donkey, with enhanced pathogenicity instead of attenuation. After 121 serial passages of EIAV_DV117_ strain on donkey leukocyte (EIAV_DLV121_), there was a progressive loss of virulence (Figure 3). EIAV_DLV121_ is an effective vaccine that can elicit protective immunity [2, 8]. This vaccine provided 85% protection for horse and 100% for donkey. However, it was costly and time consuming to isolate donkey leukocyte, so scientists successfully cultivatedEIAV_DLV121_ in fetal donkey dermal cells with 13 passages. This EIAV_FDDV13_ vaccine was as effective as EIAV_DLV121_ (Figure 3) [6]. Since 1976, this attenuated vaccine has been administered to over 70 million horses, mules, and donkeys, effectively controlling the EIAV epidemic in China.

### Virulence determinant of EIAV

Previous studies utilizing chimeric proviruses in which parental viruses are acutely virulent or avirulent have identified critical regions that affect acute virulence. These data showed that U3 regions in the viral LTR, surface envelope protein, and the accessory S2 gene strongly affects EIAV virulence [10–12]. For the Chinese EIAV live attenuated vaccine, the LTR sequences of the different generations manifested stable genetic variations and mainly occurred in the transcriptional start site, the initial base of transactivation-response element (TAR), and the pre-mRNA cleavage site at the R-U5 boundary [13]. LTR sequence diversity was increased over passages in MDMs, and the genetic distances gradually increased between these MDMs-adapted EIAV strains to the parental virulent strain EIAV_DV117_ [14]. However, LTR was not the sole determinant of virulence for EAIV [15]. LTRs of the vaccine were variable, while EIAV LTRs of virulent strains were homologous [14]. LTRs from EIAV (DLA) showed higher Tat transactivated activity than LTRs from virulent strains.

By using chimeric clones of wild-type LTR and vaccine LTR, the main differences of Tat transactivated activity were mapped to the changes of R region, rather than U3 region [16]. For S2, reverse mutation of the virulence-associated S2 gene does not cause an attenuated equine infectious anemia virus strain to regain its pathogenicity [17]. Based on a full-length infectious clone of the EIAV vaccine strain, the envelope region supports the virulence and pathogenicity of EIAV [18, 19]. Furthermore, for the transmembrane envelope (TM), there was a high frequency of GP35 in vaccine strains rather than GP45 in virulent strain. This was because the vaccine strain had a high frequency of a premature stop codon, which generated a 154-residue truncation at the C-terminus. AC-terminal truncation of the transmembrane protein of the vaccine alters its *in vitro* replication and weakens its potential pathogenicity [20]. Cytoplasmic tail truncation of EIAV Env also increased cell necrosis [21].

### Immune response induced by EIAV vaccine

**Figure 4:**
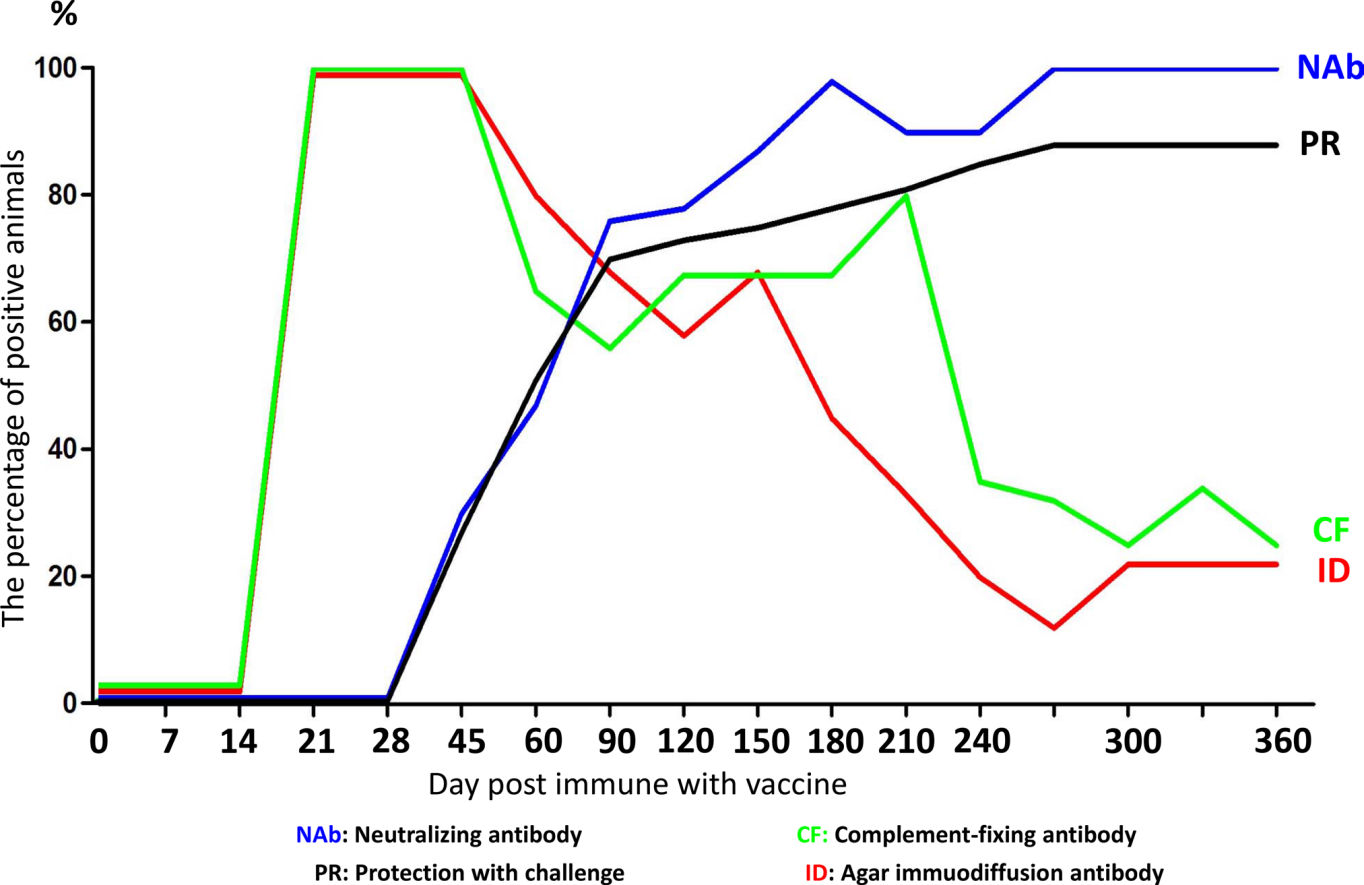
Correlation between antibody response and challenge protection The horses were vaccinated and the correlation between antibody response and challenge protection was evaluated at different days post immune with vaccine. The blue line represents neutralizing antibody; the green line represents complement-fixing antibody; the black line represents protection with challenge; the red line represents agar immuodiffusion antibody.

Vaccinated horses can produce a significant humoral immune response. Two weeks after immunization, complement-fixation (CF) antibody and AGID antibody can be detected (Figure 4). Three weeks after immunization, the CF and AGID antibody sero-conversion rate were 100% and persisted to day 45. After 45 days, CF and AGID antibody positive rate began to decline. At 360 days, this rate was decreased to 22%. Neutralizing antibodies (Nab) appeared later than CF and AGID antibody. Nab can be detected 60 days after immunization. Nab levels peaked at day180, and the Nab sero-conversion rate was 100% and sustained at a high level over a long period with little variation (Figure 4). This indicated that Nab may promote vaccine-induced immunity [22].

The EIAV-specific cellular immune response was also evaluated. CD8 positive T cells increased after immunization and induced an EIAV-specific CTL response [7, 23, 24]. The vaccine group induced high levels of Th1 cytokines, and the EIAV-specific cytokines induced by the attenuated EIAV vaccine may contribute to the protective immune response against EIA disease [25, 26].

Furthermore, vaccine-induced TLR3 activation and INF beta production were higher than in the molecular clone strains [27]. Infection of equine monocyte-derived macrophages with an attenuated equine infectious anemia virus (EIAV) strain induces a strong resistance to infection by a virulent EIAV strain [28].

### EIAV vaccine divergence and immune protection

The diverse immunogen composition of the EIAV vaccine may induce protective immunity [29–31]. Although the molecular clone strain and parental strain were replicated equally *in vitro* and *in vivo*, the molecular strain induced less protection and lower specific neutralizing production [32]. These findings indicated that diverse immunogen composition may stimulate the immune protection induced by the parental strain. *In vivo* evolution of the EIAV vaccine was investigated, and the envelope gp90 gene variation was associated with declining diversity after vaccination. This trend coincided with the maturation of immunity to EIAV [30]. Diverse immunogen composition may induce a high titer of neutralizing antibody.

### EIAV and innate restriction factors

Innate restriction factors are host factors which could intervene against every step of the viral life cycle and inhibit viral replication [33, 34]. Many innate restriction factors have antiviral functions against EIAV infection. Fv1 was first reported in the 1970s and was homologous to gag gene of endogenous retrovirus (ERV), which was as a prototypic restriction factor protecting mice against murine leukemia virus (MLV) infection [35, 36]. Fv1 could also inhibit equine infectious anemia virus (EIAV) replication, indicating that Fv1 could have a broader antiviral activity [37].

Tetherin (also known as HM1.24, BST-2, or CD317) is a type II single-pass transmembrane protein with an unusual topology, including an N-terminal cytoplasmic tail (CT), a single transmembrane domain, an extracellular domain, and a C-terminal glycosylphosphatidylinositol (GPI) membrane anchor [38]. The host restriction factor of human tetherin can block the release of enveloped viruses and inhibit viral replication. Yin et al showed that the N-terminal domain of equine tetherin was shorter than human tetherin. Equine tetherin was localized on the cell surface and strongly blocked equine infectious anemia virus (EIAV) release. Moreover, EIAV envelope protein could neutralize the antiviral activity of equine tetherin [39].

Type I interferon (IFN) and the subsequent induction of interferon-stimulated genes (ISGs) promotes resisting viral infection. Viperin, also known as RSAD2, is an endoplasmicreticulum (ER) associated multifunctional protein with broad antiviral activity. Tang et al. demonstrated that equine viperin could distort the ER, inhibit viral Gag production and/or release, Env and Receptor, leading to reduced EIAV replication [40].

Schlafen (SLFN) family proteins are divided into three groups (Groups I, II, and III) based on size and structure. All these proteins contain a specific SLFN box upstream of the AAA domain, which has ATP-binding activity. Because of the RNA/DNA helicase function of the AAA domain, SLFNs have RNA structure-modelling activity and participate in RNA metabolism [41, 42]. Codon usage-based SLFN inhibition is a novel antiviral mechanism in innate immune response. Human schlafen11 (hSLFN11) could bind to transfer (t) RNAs and inhibit the changes of the tRNA pool, which would accommodate the specific codon usage of viruses during retroviral replication [43]. Equine schlafen 11 can restrict EIAV production and replication by a codon usage-dependent mechanism [44].

Adenosine deaminases that act on RNA (ADARs) are RNA-editing enzymes that catalyze the hydrolytic C6 deamination of adenosine (A) to produce inosine (I) in double-stranded RNA substrates [45–47]. ADAR1 is one member of the ADAR family that promotes viral evolution [48, 49] and stimulates EIAV replication and infectivity. ADAR1 may even contribute to the EIAV adaptation from horses to donkeys [50, 51].

### EIAV evolution and the current control strategies

EIAV often results in a rapid, dynamic, three-stage disease process: acute, chronic, and long-term asymptomatic [52]. The initial acute phase is usually observed from 3 to 4 weeks post infection and is characterized by high levels of viremia and severe clinical symptoms. Most infected horses progress to chronic EIA, associated with repeated cycles of disease. With the irregular intervals of EIA cycle and the development of host immunity, most infected horses then become long-term asymptomatic. This makes EIAV an ideal model for understanding viral evolution in persistence and pathogenesis, providing better strategies for controlling EIA.

Because viral envelope variants were produced and selected sequentially, the virus could escape the host immune responses, leading to the cyclic nature of chronic EIA [53, 54]. Surface envelope glycoprotein gp90is the predominant site of viral variation of EIAV. Leroux et al showed that gp90 variation was independent of the number of disease cycles, viremia during chronic disease, and levels of virus replication during long-term asymptomatic infection. EIAV evolution can be associated with selection in target tissues, ongoing low levels of virus replication and be lack of abundant levels of plasma viremia [55].

The long terminal repeat (LTR) enhancer region of EIAV is one of the most variable regions in the EIAV genome [56, 57]. Up to 45% of the nucleotide positions within the LTR enhancer varied between the isolates [58]. EIAV LTR sequence variation influences the virus tropism. LTR enhancer changes are associated with EIAV adaptation in tissue culture [57]. Maury et al also indicated that due to a consequence of cell-specific selective pressures, genetic variants of EIAV LTR influenced cell tropism. However, there was no changes in the LTR enhancer over the course of a 3-year infection, implying the evolution of EIAV LTR was stable *in vivo* [59].

After using the attenuated vaccine EIAV_FDDV13,_ China has successfully controlled and most areas have eradicated EIAV. However, EIA is still prevalent worldwide, especially in France, Slovenia, Ireland, Belgium, Italy, Japan, and Brazil [60–65]. Some of China’s experiences and measures can be adopted to battle EIA. First, the summer mosquito eradication must be conducted carefully. Second, regular quarantine is necessary. Serological examination is the main method for finding and euthanizing EIA-positive horses in a timely manner. Thirdly, all the horses should be immunized with an effective vaccine. Chinese EIAV attenuated vaccine EIAV_FDDV13_ is a very effective vaccine to control EIA.

### Perspective

Developing an effective vaccine against lentivirus infections is still needed in both human and veterinarian medicine. EIA is still a severe lentivirus disease in horses of many other countries. In China, because many effective strategies were applied including the EIAV live attenuated vaccine, EIAV was successfully controlled and most areas have eradicated EIAV. While this serves as a model for controlling EIAV in other countries and battling other viral diseases, the attenuation mechanisms in these passages were not well understood. Understanding the attenuation mechanisms in the EIAV vaccine will provide further insight to controlling EIAV and other viruses.
